# Allopurinol treatment changes microglial characteristics in neonatal mice

**DOI:** 10.17912/micropub.biology.001739

**Published:** 2025-07-30

**Authors:** Rin-ichiro Teruya, Tomomi Okajima-Takahashi, Fuminori Tsuruta

**Affiliations:** 1 Doctoral Program in Biology, Degree Programs in Life and Earth Sciences, Graduate School of Science and Technology, University of Tsukuba, Tsukuba, Japan; 2 Graduate School of Life and Environmental Sciences, University of Tsukuba, University of Tsukuba, Tsukuba, Japan; 3 Master's and Doctoral Program in Biology, Institute of Life and Environmental Sciences, University of Tsukuba, University of Tsukuba, Tsukuba, Japan; 4 Master's and Doctoral Program in Neuroscience, Graduate School of Comprehensive Human Sciences, University of Tsukuba, University of Tsukuba, Tsukuba, Japan; 5 Ph.D. Program in Human Biology, School of Integrative and Global Majors, University of Tsukuba, University of Tsukuba, Tsukuba, Japan; 6 Ph.D. Program in Humanics, School of Integrative and Global Majors, University of Tsukuba, University of Tsukuba, Tsukuba, Japan

## Abstract

Microglia are resident immune cells that play crucial roles in regulating brain development. During the pre and postnatal stage, microglial morphology gradually alters by the elongation of processes and an increase in the number of branches. Previously, we reported that hypoxanthine, a key intermediate of the purine metabolism, affects the morphology of microglial cell line BV2. In this study, we show that administration of allopurinol, an inhibitor of xanthine oxidase, changes microglial morphology *in vivo* . We found that the number of branches and summed length of processes are increased in allopurinol-treated microglia in a sex-independent manner. Notably, allopurinol administration altered the number of IBA1-positive microglia in male mice. These findings suggest that purine metabolism contributes to the regulation of microglial characteristics during neonatal brain development.

**Figure 1. Allopurinol administration regulates microglial morphology:**
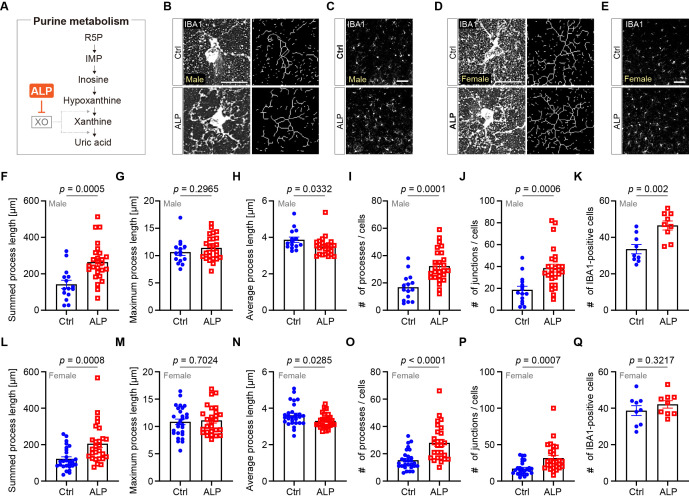
**(A)** The purine metabolic pathway. Allopurinol (ALP) inhibits xanthine oxidase (XO), which catalyzes the reaction of hypoxanthine and xanthine to uric acid. **(B)** Immunostainings showing the expression of IBA1 in either vehicle- or ALP-administrated male mice. The right panels show the binary images converted from the left panels. P14, cortical layer 2/3, Scale bar = 20 µm. **(C)** Immunostainings showing the number of IBA1-positive microglia in male mice after either vehicle- or ALP-administration. P14, cortical layer 2/3, Scale bar = 50 µm. **(D)** Immunostainings showing the expression of IBA1 in either vehicle- or ALP-administratedfemale mice. The right panels show the binary images converted from the left panels. P14, cortical layer 2/3, Scale bar = 20 µm. **(E)** Immunostainings showing the number of IBA1-positive microglia in female mice after vehicle- or ALP-administration. P14, cortical layer 2/3, Scale bar = 50 µm. **(F to J)** Quantification of microglial processes using automated skeletonized assay. Data were collected from male mice. P14, cortical layer 2/3, Ctrl; n = 3 mice, 15 cells, ALP; n = 3 mice, 26 cells. mean ± SEM, student’s *t* -test. **(K)** The quantification of the number of IBA1-positive cells in the layer 2/3 of cerebral cortex. Ctrl; n = 3 mice, 9 fields, ALP; n = 3 mice, 9 fields. mean ± SEM, student’s *t* -test. **(L to P)** Data were collected from female mice. P14, cortical layer 2/3, Ctrl; n = 3 mice, 28 cells, ALP; n = 3 mice, 27 cells. mean ± SEM, student’s *t* -test. **(Q)** The quantification of the number of IBA1-positive cells in the layer 2/3 of cerebral cortex. Ctrl; n = 3 mice, 9 fields, ALP; n = 3 mice, 9 fields. mean ± SEM, student’s *t* -test. Ctrl: vehicle (PBS).

## Description

Microglia are resident immune cells in the central nervous system. It is well established that microglia play essential roles in neurodevelopment, including synaptic pruning, debris phagocytosis, and neural circuit formation (Li and Barres, 2018; Prinz et al., 2021). Microglia originate from erythromyeloid progenitor cells (EMPs), which are derived from the yolk sac at embryonic day 7.5 (E7.5) and infiltrate the brain primordium at E9.5 through the immature circulatory system (Ginhoux et al., 2010). During this embryonic period, microglial progenitors exhibit macrophage-like morphology without distinct processes. Subsequently, postnatal microglia develop complex morphology by forming new processes and branches (Perez-Pouchoulen et al *.* , 2015). Such dramatic microglial transformation requires dynamic alterations in the cytoskeleton and lipid organization. Also, the morphological changes demand substantial energy sources, including ATP and GTP. ATP and GTP serve as essential energy sources for a wide range of cellular functions. These nucleotides are synthesized through several pathways, including mitochondrial respiration and purine metabolism. The purine metabolic pathway consists of two principal routes: the *de novo* pathway and the salvage pathway. In the *de novo* pathway, purine nucleotides are synthesized from phosphoribosyl pyrophosphate, consuming large amounts of ATP. On the other hand, the salvage pathway recycles purine byproducts, such as hypoxanthine and guanine, and produces purine nucleotides, inosine monophosphate (IMP) and guanosine monophosphate (GMP) without consuming lots of energy. In the context of high demands of energy, such as developmental stages, salvage pathway activity is important for the generation of energy source and nucleic acids (Sekine et al *.* , 2024; Tran et al *.* , 2024).

Disruption of purine metabolism can lead to various diseases due to excess uric acid production, resulting in conditions such as gout and hyperuricemia (Torres and Puig, 2007). For gout and hyperuricemia, allopurinol (ALP), a structural isomer of hypoxanthine, is a widely used treatment (Pacher et al *.* , 2006). ALP acts as a competitive inhibitor of xanthine oxidase (XO), an enzyme that catalyzes the reaction of hypoxanthine to xanthine and xanthine to uric acid ( Figure 1A ), reducing uric acid levels and alleviating symptoms. Based on these characteristics, inhibition of XO by ALP can lead to the accumulation of intracellular hypoxanthine. Our previous *in vitro* studies have suggested that treatment with hypoxanthine changes cellular morphology of microglial cell line, BV2 (Okajima et al *.* , 2020). Thus, we hypothesized that elevating hypoxanthine affects microglial morphology *in vivo* . To examine this idea, we stimulated mice with ALP intraperitoneally from postnatal day (P) 7 to P14. We then performed immunostaining for Ionized calcium-binding adapter molecule 1 (IBA1), a marker of microglia, and analyzed microglial morphology ( Figure 1B to 1E). ALP treatment increased the summed length of microglial processes in a sex-independent manner ( Figure 1F and 1L). However, the length of maximum process, which is likely to be a primary process, remained unchanged ( Figure 1G and 1M). In addition, the average length was slightly decreased ( Figure 1H and 1N). Since the summed processes were increased after ALP treatment despite little change of maximum and average length, we speculated that ALP treatment alters the number of branches. While process length is one indicator of microglial characteristics, morphological complexity reflected by the number of processes and branch junctions is another critical feature. ALP treatment significantly increased both the number of processes ( Figure 1I and 1O) and junctions ( Figure 1J and 1P) in male and female mice. These findings suggest that inhibition of XO activity enhances the morphological complexity of microglia during postnatal development.

Previous studies have reported that microglia occupy non-overlapping territories in the brain (Barry-Carroll et al *.* , 2023). This observation suggests that morphological changes may be associated with alteration in cell number or spatial distribution. Additionally, purine metabolism is required for DNA synthesis, and its disruption impairs cell proliferation (Diehl et al *.* , 2022). We therefore examined whether ALP administration influences the number of microglia. Quantification of IBA1-positive microglia revealed a trend toward increased the number in both sexes; however, the significant increase in male mice was observed by ALP treatment ( Figure 1K and 1Q). These results suggest that the effects of ALP on microglial development may exhibit sex-specific differences.

Our findings demonstrate that ALP treatment, commonly used for managing chronic gout and hyperuricemia, can influence microglial morphology during postnatal development. Notably, microglia undergo significant morphological changes during this period, transitioning from amoeboid-like forms to highly ramified forms. We observed that ALP administration increased microglial complexity, raising the possibility that ALP has broader effects beyond its established role in suppressing uric acid production. Previous *in vitro* studies from our group showed that hypoxanthine promotes microglial process elongation. Although we did not directly measure hypoxanthine levels, it is likely that ALP treatment causes hypoxanthine accumulation involved in microglial morphological changes. On the other hand, ALP may exert its effects through other mechanisms. XO generates reactive oxygen species, including hydrogen peroxide and superoxide anion, as byproducts of its enzymatic activity (Berry and Hare, 2004; Cantu-Medellin and Kelley, 2013). ALP has been reported to reduce oxidative stress by inhibiting xanthine oxidase enzymatic activity (Farquharson et al., 2002; Kang et al., 2006). Given the established link between oxidative stress and altered microglial characteristics (Kim et al., 2010), it is plausible that reduced oxidative stress contributes to the morphological changes observed with ALP treatment.

Notably, we observed a sex-specific effect on the number of IBA1-positive cells, with a significant increase observed only in male mice. However, ALP treatment affects Iba1 expression levels in a sex-independent manner, which may confound interpretation of microglial cell number. Sex differences in purine metabolism have been previously reported. For example, Lesch-Nyhan syndrome, which is a purine metabolism disorder caused by mutations in the X-linked gene HPRT1 (hypoxanthine-guanine phosphoribosyltransferase 1), primarily affects males (Lesch and Nyhan, 1964). Interestingly, microglial morphology and territory formation are altered by both brain region and sex (Grabert et al *.* , 2016; Colombo et al *.* , 2022; Barry-Carroll et al *.* , 2023). Among these differences in territory formation are pronounced in the cerebral cortex. In contrast, regional differences in microglial morphology and gene expression are also observed in areas such as the cerebellum during developmental and aged stages. In this study, we focused on layer 2/3 of the cerebral cortex; however, it is likely that microglia in other brain regions may also be regulated by purine metabolism, and further studies will be needed.

Taken together, our study suggests that purine metabolism may play a crucial role in shaping microglial morphology during early postnatal development. These findings also underscore the importance of considering metabolic and sex-dependent factors in the study of microglial biology.

## Methods

**Mice**

The C57BL/6J mice were obtained from Japan SLC, Inc. (Shizuoka, Japan). All mice were maintained under a 12-hour light/12-hour dark cycle (lights on at 8:00 AM) in a temperature- and humidity-controlled environment. Mice were administrated by ALP (100 mg/kg) （A0907, Tokyo chemical industry, Japan）intraperitoneally every 24 h from P7 to P14.

**Immunohistochemistry**

The mice brains were perfused with PBS and fixed overnight in 4% paraformaldehyde (PFA) in PBS. After infiltration with 30% sucrose in PBS, the samples were embedded in Tissue-Tek OTC compound (Sakura Finetek, Tokyo, Japan) and sectioned at a thickness of 50 µm by a cryostat (CM1950, Leica Biosystems, Wetzlar, Germany). Free-floating sections were permeabilized and blocked with PBS containing 0.25% Triton X-100 and 5% bovine serum albumin (BSA). Primary antibodies were incubated in PBS containing 5% BSA for 3 days [goat anti-IBA11 antibody (Abcam, Cat# ab5076, 1:200)] at 4°C. Brain sections were washed with PBS and incubated with Donkey anti-goat IgG (H+L) antibody, Alexa Fluor 488 (Abcam, Cat# ab150129, 1:1000) for 1 hour at room temperature. Samples were mounted in VECTASHIELD Mounting Medium (Vector Laboratories). Tissue specimens were observed using the confocal laser scanning microscope (LSM700, Carl Zeiss) with ×20 (Plan-Apochromat ×20/0.8 M27) and×63 (Plan-Apochromat ×63/1.4 Oil DIC M27) objectives. Diode excitation lasers (Diode 488) were operated and directed to a photomultiplier tube (LSM T-PMT, Carl Zeiss) through a series of bandpass filters. Z stack images (interval, 1 µm per image) were acquired using ZEN software (Carl Zeiss). The microglial skeletal assay was assessed by the ImageJ software.

**Statistical analysis and image processing**

The statistical analyses were calculated by using GraphPad Prism (GraphPad Software). All image processing was conducted by FIJI Image J 2.1.0/1.53c. The number of cells were quantified by Fiji/ImageJ software. No data were excluded from the statistical analysis. For comparisons between two groups, we used Student’s *t* -test. All representative microscopic images shown in the figures reflect at least three biological replicates.

&nbsp;
